# Interaction between tick and host microbiotas: a four-step waltz

**DOI:** 10.1186/s13071-026-07308-1

**Published:** 2026-02-18

**Authors:** F. Baquer, A. Grillon

**Affiliations:** 1 Institut de Bactériologie, Université de Strasbourg, UR3073, 67000 Strasbourg, France; 2 Laboratory of Bacteriology, Hôpitaux Universitaires de Strasbourg, 67000 Strasbourg, France; 3 French National Reference Center for Borrelia, Hôpitaux Universitaires de Strasbourg, 67000 Strasbourg, France

**Keywords:** Tick-borne diseases, Skin microbiota, Tick microbiome, Endosymbionts, Vector competence, Immune priming, Dysbiosis, Disease prevention

## Abstract

Tick-borne diseases represent a growing public health concern worldwide, yet the microbial factors that govern pathogen transmission remain incompletely understood. Over the past decade, high-throughput metagenomics and functional studies have revealed that two distinct microbial communities—the vertebrate host’s skin microbiota and the tick’s own microbiome—act synergistically as key modulators of pathogen acquisition, persistence within the vector, and successful transmission to the vertebrate host. At the feeding site, the skin microbiota orchestrates local cutaneous immunity, influences inflammatory responses, and can either hinder or inadvertently facilitate dermal establishment of tick-borne pathogens such as *Borrelia burgdorferi* sensu lato (s.l.), *Anaplasma phagocytophilum*, *Rickettsia* species, *Babesia* spp., and tick-borne encephalitis virus. Tick feeding itself induces rapid and sometimes long-lasting dysbiosis of the skin microbial community, creating temporal windows of vulnerability for pathogen invasion. Concurrently, within the tick vector, a core set of endosymbiotic bacteria, including *Rickettsia buchneri*, *Midichloria mitochondrii*, *Coxiella*-like, and *Francisella*-like endosymbionts, engage in complex mutualistic, competitive, and facilitative interactions. These symbionts regulate vector competence through nutrient provisioning (especially B-vitamins), direct competition for niche space, and immune priming or suppression of the tick’s innate immune system. Such interactions ultimately determine the maintenance, abundance, and transmissibility of tick-borne pathogens. By integrating these dual host–vector microbiome perspectives in a comprehensive review, we highlight emerging mechanistic insights into transmission ecology and biologically grounded targets for the prevention and control of tick-borne diseases, including anti-microbiota vaccines and paratransgenic and microbiome-based approaches.

**Graphical Abstract:**

**Figure:**
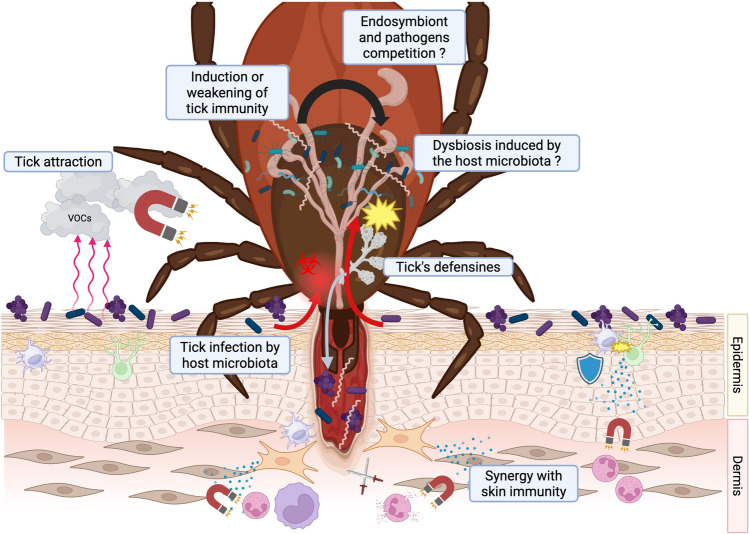

## Background

The investigation of microbiota has been a prominent research focus for many years. Although the gut microbiota has been the primary subject of study due to its extensive applications, the skin microbiota is increasingly recognized as equally significant. Its involvement in healing and inflammatory skin diseases makes it a significant contributor to human health [1, 2].

Ticks are major vectors of microorganisms potentially affecting human and animal health. Vector systems, traditionally considered to be three-actor systems, have recently been reconceptualized as more complex frameworks, with the microbiota now recognized as a fourth integral component. First suspected to influence the presence of arboviruses in Culicoides insects [3], the insect intestinal microbiota—predominantly composed of Gram-negative bacteria—was later identified as essential in the establishment of *Plasmodium*, the causative agent of human malaria [4]. Later on, a similar observation was realized in *Ixodes* ticks colonized by *Borrelia burgdorferi* sensu stricto (s.s.), the etiological agent of Lyme borreliosis [5].

The skin interface of the vertebrate host and its microbiota has emerged as a key regulator of cutaneous inflammation [6]. Given its critical role in vector-borne diseases [7], the skin microbiota is likely to contribute to immunomodulation during the pathogen transmission, especially in hard ticks that perform a telmophagous bite and remain deeply embedded within the skin for several days [8]. Given that ixodid ticks spend most of their lives in microorganism-rich environments, are in close contact with the skin microbiota during their blood meal on the vertebrate host, and are blood pool feeders for several days [9], identifying the different levels of interaction between pathogen, host, and tick microbiota should improve our understanding of pathogen development within the tick and pathogen transmission to the vertebrate host.

This review delineates four distinct facets of the intricate interplay between the vertebrate host’s cutaneous microbiome and the tick microbiome in tick-borne pathogen transmission—a metaphorical “waltz in four time”: (1) the tick microbiome deeply dictates overall vector competence, encompassing pathogen acquisition, persistence, and transmission efficacy; (2) the host skin microbiome modulates vector attraction through volatile chemical cues; (3) tick attachment and feeding trigger dysbiosis at the bite site, profoundly reshaping skin microbiomes during wound formation, but the tick also ingests large amounts of the host's microbiome; and (4) this local perturbation governs interactions between the cutaneous microbiome and inoculated tick-borne pathogens, influencing their initial establishment and subsequent dissemination.

### The tick microbiome, a major driver of vector competence

The tick microbiota refers to the ensemble of microorganisms associated with the tick, including three main ecological and functional categories that differ markedly in their origin, transmission mode, stability, and interactions with the host and pathogens:

1. **Core endosymbionts**: obligate or near-obligate vertically transmitted bacteria (e.g., *Candidatus* Midichloria mitochondrii in *Ixodes ricinus*, *Spiroplasma ixodetis* in *Ixodes frontalis*, *Rickettsia buchneri* in *Ixodes scapularis*, Coxiella-like endosymbionts in *Rhipicephalus* spp., Francisella-like endosymbionts in *Amblyomma* spp.) [10, 11]. These symbionts are maintained across tick generations through transovarial transmission and typically show high prevalence and low genetic diversity within tick populations [11–13].
2. **Facultative / transient microbiota**: microorganisms acquired horizontally during the tick’s free-living stages (environmental bacteria, e.g., *Pseudomonas, Sphingomonas*), during blood feeding (host skin derived) or can be brought by parasitic wasps (Arsenophonus, Wolbachia spp.). Their composition is more variable and depends on ecological factors and recent feeding history [11].
3. **Pathogenic or opportunistic microorganisms**: tick-borne pathogens (e.g., *Borrelia burgdorferi* sensu lato, *Anaplasma phagocytophilum*, *Rickettsia* spp. of the spotted fever group) or other potentially pathogenic microbes that may be transiently present [10, 11, 14, 15].

The observed patterns of tick colonization among these three categories are consistent with ecological assembly frameworks integrating stochasticity, deterministic processes, priority effects, keystone taxa, and network stability, as detailed by Obregon et al. [16]. On the one hand, core endosymbionts are transmitted transovarially and therefore follow a conserved and deterministic distribution within a tick species. On the other hand, transient or facultative microorganisms and pathogens seem to follow a stochastic distribution, which is influenced by multiple factors, such as tick stasis, geographical location, climate, and available hosts. This microbial community assembly represents an emerging challenge in identifying the various factors involved.

When considered at the organ level, the tick microbiota exhibits marked heterogeneity, with distinct microbial communities associated with the midgut, ovaries, salivary glands, and cuticle [17].

Midgut microbiota is highly dependent on feeding status: as high diversity is observed in unfed ticks, a dramatic decrease is noticed in fed ticks [18]. Most of the tick gut microbiome is made up of endosymbionts. Rickettsiales are essentially represented by *Rickettsiella* endosymbionts, nonpathogenic *Rickettsia*-like endosymbiont (LE) [19, 20], *Coxiella*-LE [19, 21–23], and more rarely by pathogenic *Rickettsia* belonging to the spotted fever group or *Coxiella burnetii* [24]. Similarly, *Francisella* are mainly *Francisella*-LE [25].

Composition of the midgut microbiota also varies according to tick species. Midgut microbiota of *I. ricinus*, mainly composed of noncultivable bacteria, harbors Proteobacteria phylum, although other phyla such as Firmicutes, Bacteroidetes, Actinobacteria, Spirochetes, and Tenericutes have been reported [26]. The abundance of these phyla varies between ticks and collection areas, suggesting a prominent role of environmental factors [26]. At the genus level, *Lariskella* is more common in *Ixodes persulcatus* [18], and *Francisella* are very abundant in *Amblyomma americanum* [27]. Typically, the predominant species is the essential symbiont associated with the tick species: *Candidatus* Midichloria mitochondrii for *Ixodes ricinus*, *Rickettsia buchneri* for *Ixodes scapularis*, *Coxiella*-LE or *Francisella*-LE for *Rhipicephalus* spp. or *Amblyomma maculatum*, respectively [28]. The gut microbiome also seems to be enriched by host skin bacteria during the blood meal (parasitic phase) or by environmental bacteria during the free life of the tick [18, 29]. Among these bacteria, several notable groups stand out. Some genera are present in low abundance but with a high frequency, such as *Streptococcus* and *Staphylococcus*, which probably originate from the skin of mammalian hosts, and *Ralstonia*, *Pelomonas*, *Pseudomonas*, *Stenotrophomonas*, or *Achromobacter*, which are common environmental bacteria [18, 26, 27, 29]. Human pathogenic bacteria such as *Borrelia myamotoi* or *Borrelia burgdorferi* s.l. can sometimes be found in very high abundance in the tick’s midgut [17]. *Borrelia burgdorferi* s.l. generally appears to be more abundant in the midgut than in the salivary glands [17] due to the interaction between the *Borrelia* surface lipoprotein OspA and its receptor TROSPA expressed by the digestive epithelial cells of the tick [30].

Concerning the salivary gland microbiota, it essentially depends on the tick nutritional status, as for the midgut. Fully fed ticks show more diversity than partially fed or unfed ticks [18, 29]. Potential pathogens are occasionally present in the tick salivary glands from fed ticks such as *Neoehrlichia mikurensis*, *Anaplasma* spp., and *Rickettsia*, or viruses such as tick-borne encephalitis virus and Crimean–Congo hemorrhagic fever virus [17, 31–34].

Concerning the tick cuticle, given the tick lifestyle—alternating between free-living in humus and vegetation, and a parasite anchored to the host skin—it likely modifies the the cuticle-associated microbiota. As many tick DNA extraction protocols rely on alcohol washing and rinsing of the whole tick, it is not surprising that the cuticle microbiota has been little investigated. In addition, the low diversity of the salivary glands or digestive microbiota and the predominance of certain genera mask the diversity of the cuticle microbiome in metagenomic studies on whole ticks [18]. The most recent advances on the tick cuticle microbiota come from Wiesinger et al., who dissected ticks to study the salivary gland and the gut microbiota, as well as the remaining parts of the tick, which includes the cuticle [35]. Shannon indices show a higher alpha diversity than in the midgut or salivary glands, and a higher beta diversity. *Pseudomonadaceae*, *Shingomonadaceae*, *Beijerinckiaceae*, and *Microbacteriaceae* families can be identified, not surprisingly from environmental origin.

Ovaries are characterized by a remarkably stable microbial community, showing low diversity regardless of feeding status. Ovary microbiota is vertically transmitted to offspring and is mainly composed of *Coxiella*-LE or *Candidatus* *Midichloria mitochondrii*, *Rickettsia* spp., *Francisella* spp., and *Wolbachia* spp. [18].

Ticks are obligate blood feeders, that is, their diet depends exclusively on the blood of parasitized vertebrate hosts. Endosymbionts therefore play an essential role in providing the deficient nutrients, such as B vitamins supplied by *Coxiella*-LE [36, 37], *Francisella*-LE [38], *Rickettsia*-LE [39], and *Midichloria* [40]. The reduction in symbionts supplying B vitamins alters the development of nymphs into adult stages [38]. However, some symbionts contribute more importantly than the others. *Coxiella*-LE also produces chorismate, a tryptophan precursor involved in serotonin synthesis, involved in the tick blood feeding activity [41], and it induces the expression of numerous genes required for digestion [42]. Their removal reduces engorgement, molting, and capacity of female ticks to produce eggs [11]. *Midichloria* carry genes associated with major metabolic pathways, such as the Krebs cycle in mitochondria, or gluconeogenesis and glycolysis. ATP/ADP (adenosine tri/diphosphate) transporters are also encoded in these bacteria, suggesting a possible source of ATP for the tick [11, 40].

Accumulating evidence suggests that the tick microbiota may play a role in shaping vector competence for bacterial pathogens (Table 1). Some symbionts are negatively correlated with abundance of tick-borne pathogens. [43–45]. Conversely, some of them are associated with other tick-borne pathogens[46]. For example, nonpathogenic endosymbionts *Rickettsia* or *Francisella* and their pathogenic parents are only exceptionally associated, suggesting potential competitive exclusion [46].

**Table 1:** Main interactions in the tick microbiome and their impacts on vector competence

Actor	Pathogen involved	Type of interaction	Effect on vector competence	Evidence	References
Nonpathogenic *Rickettsia*-LE or *Francisella*-LE	Pathogenic *Rickettsia* spp. or *Francisella* spp.	Negative (competitive exclusion)	Rare co-occurrence with related pathogens; reduces abundance and transstadial transmission	No (suggested by exceptional associations)	[46]
*Anaplasma phagocytophilum*	*Borrelia burgdorferi* s.l.	Negative (microbiota depletion)	Induces antifreeze glycoprotein → depletes midgut microbiota → downregulates JAK-STAT → disrupts peritrophic membrane, favoring *A. phagocytophilum* but limiting *Borrelia* persistence	Yes (mechanism demonstrated)	[48, 49]
*Midichloria mitochondrii*	*Neoehrlichia mikurensis, Borrelia burgdorferi* s.l.	Positive (facilitation via immune tolerance and stress tolerance)	Induces immune tolerance and stress tolerance favoring colonization and persistence; stimulates sylvatic cycle and contributes to Lyme disease risk	Yes (positive correlations and mechanisms demonstrated)	[43, 53, 54]
*Borrelia burgdorferi* s.l.		Positive (facilitation via microbiota modulation)	*Pixr* expression induced by *B. burgdorferi* → modulates gut microbiota (inhibits Gram-positive biofilm formation); enhances *Borrelia* colonization in midgut, increasing acquisition/persistence	Yes (mechanism demonstrated)	[48]
*Midgut microbiota*	*Borrelia burgdorferi* s.l.	Positive	Tick midgut microbiota limits the accumulation of lysin, responsible for DefMT6 induction, a tick defensine that exhibits borreliacidal effect	Yes (mechanism demonstrated)	[51]

Negative interactions between symbionts and pathogens are probably explained by a combination of competition and manipulation of the tick’s immunity [47]. The Toll and IMD pathways are responsible for the production of antimicrobial peptides that make the tick’s midgut an environment that is unlikely to be colonized by exogenous species. *Anaplasma phagocytophilum* induces the production of antifreeze glycoprotein to deplete the tick’s midgut microbiota. This depletion is responsible for the underactivation of the JAK-STAT pathway (Janus Kinase and Signal Transducers and Activators of Transcription proteins), which is necessary for the integrity of the peritrophic membrane. This increases its ability to invade tick cells but also limits the abundance of *Borrelia burgdorferi* s.l., which is closely dependent on this membrane [48, 49]. *Borrelia* also uses a similar method to limit competition in its niche by promoting the expression of the *pixr* protein in *I. scapularis*, which is involved in larval molting and acts as a powerful biofilm inhibitor [48]. Similar observations have been described by Hamiltone et al., suggesting that infection with *B. afzelii* can significantly influence the bacterial microbiome of *I. ricinus* nymphs [50].

Recent studies reveal additional layers of functional integration. Anti-microbiota vaccination disrupts endosymbiont communities, leading to lysine accumulation in tick hemolymph. This metabolite acts as an upstream signal that upregulates defensin expression (e.g., DefMT6) and impairs *Borrelia burgdorferi* colonization, establishing a direct microbiota–metabolite–immunity axis [51].

Conversely, *Midichloria mitochondri* increases stress tolerance and induces a form of immune tolerance that favors certain pathogens such as *N. mikurensis* and *B. burgdorferi* s.l. [43, 52, 53]. Recent field and experimental studies have further elucidated the facilitative role of *M. mitochondrii* in *Borrelia burgdorferi* s.l. dynamics [53]. Infection with *M. mitochondrii* significantly enhances the acquisition of *B. burgdorferi* s.l. by feeding larvae on naturally infected hosts, and positively correlates with higher infection rates in questing nymphs across geographic locations. This suggests that the endosymbiont promotes both pathogen uptake and persistence in immature stages, thereby stimulating the sylvatic cycle and contributing to elevated Lyme disease risk in endemic areas [54].

Beyond pairwise interactions, community-level network analyses demonstrate that *Borrelia* infection reshapes microbial co-occurrence patterns. Deviations from these pathogen-induced network structures (e.g., anti-microbiota vaccine) are associated with reduced pathogen loads, underscoring the role of holistic community dynamics in vector competence [55].

Moreover, as shown in the sandfly, the insect involved in the transmission of the parasite *Leishmania* spp., the insect microbiota can also facilitate the transmission of pathogen to the vertebrate host [56]. It has been shown that prior treatment of *Leishmania* infected-sandflies with antibiotics impairs the development of the parasite within the skin of the vertebrate host [57].

To date, no symbiotic bacteria have been described as modulators of pathogenic bacteria during the tick bite.

While the tick’s internal microbiome profoundly influences pathogen persistence once the blood meal is ingested, the transmission process begins much earlier at the skin surface, where chemical cues from the vertebrate microbiota can already determine whether a tick will attach and feed.

### Skin microbiota and tick attraction

The skin is an essential barrier for protection from the environment. As opposed to oropharyngeal or digestive microbiota, environmental conditions vary drastically according to anatomical areas [58]. Among those are temperature, which is lower at the extremities, and humidity, which is higher at axillary and inguinal folds [59, 60]. Several studies have analyzed the composition of the skin microbiome according to specific sites on the skin, which have been divided into dry, sebaceous, and humid areas [6]. Lipophilic bacteria, such as *Propionibacterium* and *Cutibacterium*, are preferentially found on the sebaceous areas: face, back, and manubrium [6]. *Corynebacterium* and *Staphylococcus*, which prefer humidity, are mainly present in areas with a high concentration of apocrine and eccrine glands such as axillary, popliteal, antecubital fossa, and sole of the foot [6]. Finally, dry areas exhibit a high bacterial diversity [6]. Among fungal communities, *Malassezia* spp. predominate on human skin, and a wide variety of viruses can also be encountered [61].

While the composition of the skin microbiota is relatively stable in adulthood [62], it varies greatly during childhood and with aging.

For several decades, host skin microbiota has long been linked to the attractiveness of anthropophilic mosquitoes [63]. This attraction relies on volatile organic compounds (VOC), and most of them derived from the metabolic activity of skin bacteria [63]. Presence of vector-borne pathogens can increase host attractiveness for their own competent vectors. *Plasmodium* infected host, for example, becomes more attractive to mosquitoes [64, 65].

In ticks, chemical compounds are detected by sensory neurons in Haller’s organ [66]. While many volatile compounds have been described as attractants or repellents in animals, few are shared with humans. One of the major attractors shared with all hematophagous arthropods is CO_2_ [67]. Other notable attractants include nonanal and 1-octen-3-ol for *Amblyomma cajennense*, or acetone and NO for *Ixodes ricinus* and *Rhipicephalus sanguineus* [67, 68]. Some of these VOCs trigger opposite effects between mosquitoes and ticks, or between tick species themselves [69]. This could partially explain the observed host preference among tick species. As observed with *Plasmodium-*infected hosts, it seems that host infection by a tick-borne pathogen modify attractiveness for their own competent vectors. Thus, bank vole infected by *Borrelia afzelii* increases tick attractiveness [70]. While the precise mechanism behind these variations in attractiveness is currently unknown, it is conceivable that the host infection subtly modifies the skin microbiota or its metabolism to better attract arthropod vectors. This mutual attractiveness of infected hosts for vectors, and vectors for vertebrate hosts, could facilitate the maintenance of vector-borne diseases in the ecosystems.

### Skin microbiome during skin disruption

When their niches are disrupted, microbial communities change in structure and function and favor opportunist infections [58].

Tick bite can be compared with a mechanical skin injury, leading to an inflammatory response, although the saliva prevents healing. Tick saliva, rich in numerous effectors, dissociates fibroblasts and exerts a powerful antiinflammatory effect [71, 72].

During injury, the microbiota plays a role in healing by stimulating the secretion of CXCL10, which attracts neutrophils, helping to prevent infection and recruit plasmacytoid dendritic cells. The latter secrete interferon gamma, which stimulates fibroblasts and macrophages to repair the wound [73]. Certain commensal species, such as *S. epidermidis*, also prevent inflammation from becoming harmful and support angiogenesis, keratinocyte proliferation, and extracellular matrix regeneration [74].

However, during tick blood meal, composition of the cutaneous microbiota is progressively altered, and tends to be more similar to the tick’s midgut microbiota [45], although it appears that bacteria from the skin microbiota penetrate deeply into the feeding pit [75]. Interspecies interactions between tick’s gut and host skin microbiota and their potential effects on inflammation are currently unknown. Lastly, as tick saliva possesses strong antiinflammatory and immunomodulatory activities [72], description of all these interactions is still poorly documented and constitutes a great challenge for future research.

These disruptions not only alter the cutaneous microbial landscape, but also bring the host’s microbiota into direct contact with the tick’s midgut during the prolonged blood meal, raising the question of how these two microbial worlds interact.

### How does skin microbiota interact with ticks?

The interaction between skin microbiota and ticks is a little-studied subject. The close contact between the skin and the tick during the blood meal could involve the transmission of certain bacteria from the host to the tick. Although the tick’s way of life implies this regular contact, it seems that interactions can sometimes be conflictual. Some bacterial species from the host skin, such as Staphylococci, exhibit deleterious effect on ticks. *S. saprophyticus*, *S. xylosus*, *S. shinii*, and *S. succinus* contribute to deadly infections in *Rhipicephalus microplus* [76, 77]. This harmful effect does not seem to be limited to this tick species, as the tick *Ixodes scapularis* has developed an original bacterial toxin (Dae2) against *S. epidermidis* to enhance its survival [78]. Dae2, acquired anciently from gut-associated bacteria, was shown to control invading bacteria, either pathogenic such as *Borrelia* or host skin commensal in *Ixodes* tick [78]. Host-derived factors ingested during feeding, such as interferon-γ, can induce Dae2 peptide expression in the tick, thereby limiting pathogen proliferation [79]. Concerning *Ixodes ricinus*, several defensins and antimicrobial peptides have been characterized. Defensins 1 and 8 exhibit a broad spectrum of activity against Gram-positive bacteria of the skin microbiota, such as *Staphylococcus aureus* and *Staphylococcus epidermidis*, but no activity on Gram-negative bacteria [80]. These mechanisms appear sufficient to maintain the stability of the tick’s digestive microbiota, even following the introduction of exogenous bacteria [26]. This mechanism could also explain the relatively sparse microbial community observed in ticks [47].

Gram-negative bacteria that can transiently be found on human skin, such as *Serratia marcescens*, can also act as pathogens for *Rhipicephalus* ticks [81]. However, the precise effect of these bacterial infections on the tick biology is currently unknown.

Beyond these direct interactions, the skin microbiota also modulates the local environment in which tick-borne pathogens establish infection, influencing both innate responses and pathogen persistence at the bite site.

### Skin microbiota and vector-borne pathogens

**Fig. 1:**
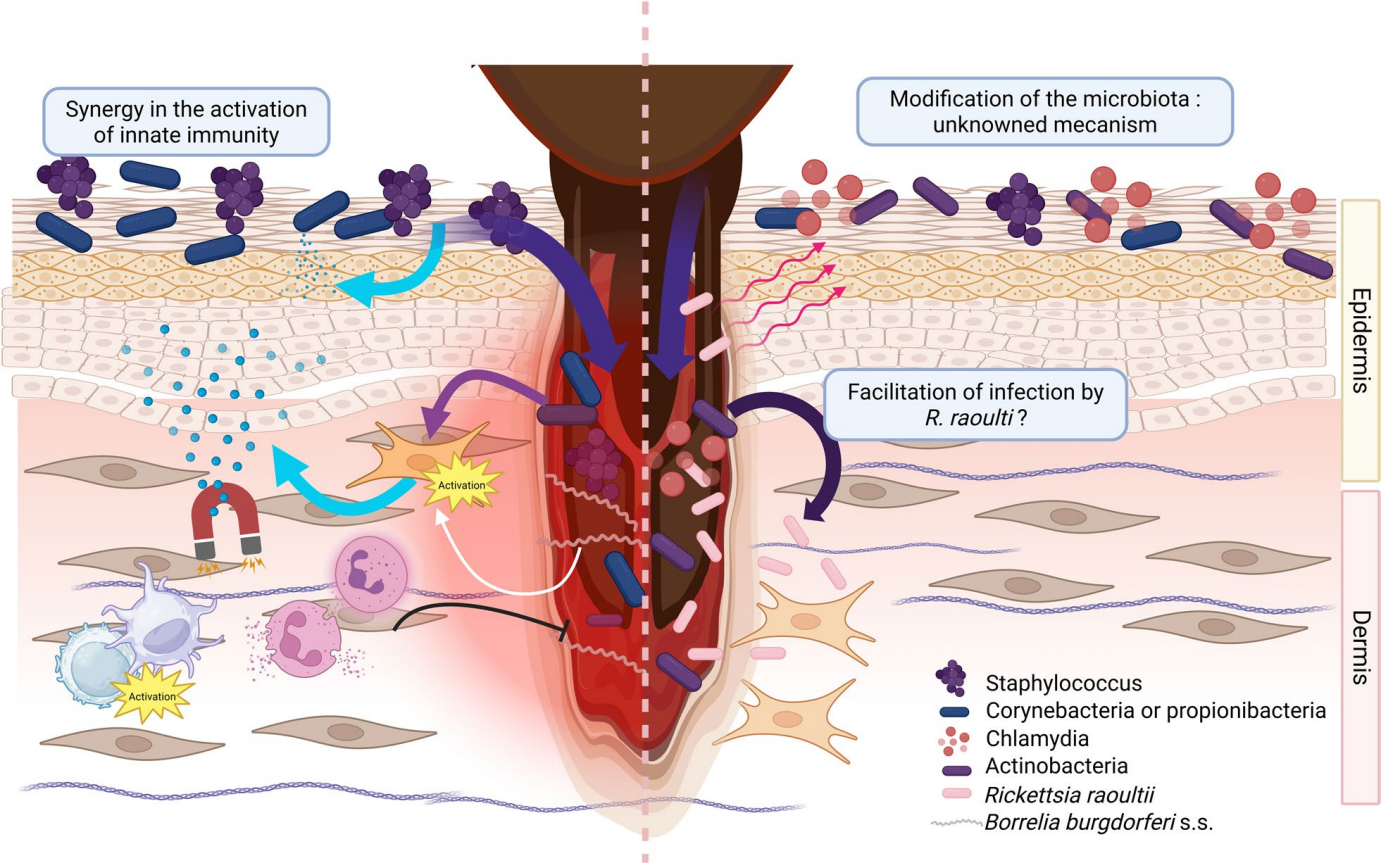
Host–microbiota relationship during tick bites. Example of infection with Borrelia and Rickettsia raoulti. **On the left**: *Borrelia burgdorferi *s.s. triggers the production of interleukin IL-8, CXCL-1, MCP-1 (monocyte chemoattractant protein 1), and hBD-2 (human beta-defensin 2) by fibroblasts in the dermis and undifferentiated keratinocytes present in the deep layers of the epidermis. This expression is potentiated by the skin microbiota (*S. epidermidis*, C. striatum, C. acnes). These compounds have chemoattractive properties on neutrophils and monocytes, facilitating the resolution of the infection. **On the right**: The bite of a tick carrier of *Rickettsia raoultii* induces a change in the host’s skin microbiota. Actinobacteria, which become more prevalent, have intense metabolic activity that could facilitate infection of the host by *R. raoultii*. Created with BioRender.com

Skin is a strategic organ in vector-borne diseases as the site of transmission, multiplication, and persistence of pathogens, such as *Borrelia* [82]. Few data are available on the influence of skin microbiome on tick-borne pathogen transmission. However, it has been suggested that skin microbiota could be impacted by *Rickettsia raoultii* infected tick-bite in a mouse model [83]. The skin microbiota appeared destabilized, with the emergence of bacterial genera such as Chlamydiae and Actinobacteria. This microbial switch appears to be associated with significant stimulation of metabolic pathways, focal adhesion, and phagosomes in Actinobacteria, which could promote infection by *R. raoultii* (Fig. 1). Regardless of the infectious status of the bite, the authors also noted alterations in the transcriptional activity of the skin microbiota, which they attributed to a reaction to tick saliva. These findings are consistent with observations of the cutaneous microbiota of tick-bitten mice, which reveal profound changes in microbial diversity following the tick bite, up to 10 days [45].

In Lyme disease, the co-incubation of molecules secreted by the skin microbiota with *B. burgdorferi* s.s. appears to increase chemokine expression by resident skin cells, thus promoting a rapid innate response at the onset of *Borrelia* infection [84] (Fig. 1).

Additionally, skin bacteria produce killing effectors such as bacteriocins, which are small peptides or proteins with antibacterial properties [85]. Their properties and activity spectra have already proved to be interesting tools in the fight against pathogens transiently hosted on the skin, such as *S. aureus* [86, 87]. As knowledge about them expands rapidly, with new molecules being continuously described, these bacteriocins could be also involved in cutaneous defense against tick-borne pathogens [86].

Finally, in vector-borne diseases, the role of skin microbiota on adaptative immune system education has been mostly studied on mice models. Although the skin microbiota is not required for the existence of immunes cells residing in the skin, it acts as a major adjuvant to the host’s immune system. Naik et al. have shown that axenic mice have a deficient inflammation during *Leishmania* inoculation, compared with pathogen-free mice. Introduction of *S. epidermidis* was subsequently sufficient to restore an effective immunity [88]. Later studies confirmed that microbiota exposure leads to cutaneous homing of various T cells [89–93], and subsequent studies suggested an educational role of microbiota, mainly on Th1 and Th17, as well as on the humoral response [94, 95]. These interactions involve a rapid distinction between healthy microbiota and invasive microorganisms by dendritic cells.

Commensal skin colonization therefore appears essential to cutaneous immunity development against vector-borne pathogens.

## Conclusions

**Fig. 2:**
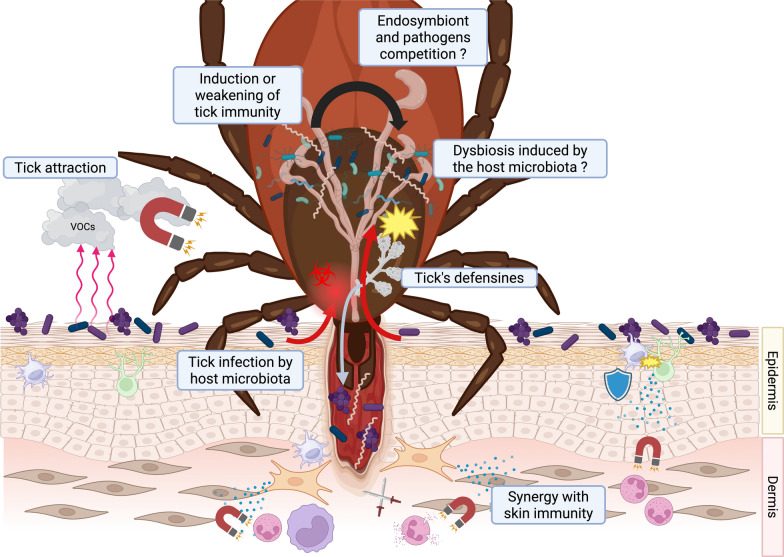
Role of skin microbiota in the transmission of tick-borne disease. Created with BioRender.com

The skin microbiota is emerging as a key player in the complex interactions between the host, the tick, and tick-borne pathogens (Fig. 2). By regulating local immunity, contributing to wound healing, and maintaining cutaneous homeostasis, it shapes the microenvironment in which pathogen transmission occurs. Conversely, disruptions of this equilibrium—whether resulting from dysbiosis induced by external factors (cosmetics, antiseptics, environmental conditions) or from developmental and age-related changes, may compromise cutaneous defenses and facilitate infection.

In parallel, the skin microbiota may represent a promising target for novel preventive or therapeutic strategies. Modulating microbial composition to reduce host attractiveness, enhancing local immune response, or developing approaches that disrupt the tick microbiota—such as vaccines directed against tick symbionts—are emerging as potential avenues to reduce pathogen transmission. In this field, the use of probiotics and postbiotics, as previously demonstrated in the promotion of wound repair, may represent a potential domain of interest [96].

Among the emerging approaches, paratransgenesis stands out as a particularly innovative strategy. By genetically engineering native tick endosymbionts such as *Rickettsia buchneri*, *Candidatus* *Midichloria mitochondrii*, or *Coxiella*-LE to express antimicrobial peptides or pathogen-blocking molecules, this method transforms harmless symbionts into targeted “biological weapons” against *Borrelia*, *Anaplasma*, or other tick-borne pathogens [97]. Thanks to their obligate vertical transmission and high prevalence in natural tick populations, these modified symbionts could achieve stable, long-term reduction in vector competence following targeted releases in endemic areas.

Antimicrobiota vaccination represents a complementary strategy to paratransgenesis. As proposed by Cabezas-Cruz et al. [98], targeting tick microbiota by cross-species host immunization could led to restructuring tick microbial communities, inducing metabolic shifts (lysine accumulation), enhancing defensin-mediated immunity [79], and reducing pathogen colonization [51, 55]. This approach is promising but currently remains conceptual.

Altogether, integrating the microbiota as a fourth component of the vectorial system, alongside the host, the tick, and the pathogen, is essential for a comprehensive understanding of tick-borne diseases. Further interdisciplinary research is required to elucidate the underlying mechanisms of these interactions and to assess their potential exploitation for innovative control strategies [55].

## Data Availability

Data supporting the main conclusions of this study are included in the manuscript.

## Abbreviations

s.l.: Sensu lato
s.s.: Sensu stricto
LE: Like endosymbiont
VOC: Volatile organic compounds
